# Chemokine Receptor Ccr6 Deficiency Alters Hepatic Inflammatory Cell Recruitment and Promotes Liver Inflammation and Fibrosis

**DOI:** 10.1371/journal.pone.0145147

**Published:** 2015-12-21

**Authors:** Silvia Affò, Daniel Rodrigo-Torres, Delia Blaya, Oriol Morales-Ibanez, Mar Coll, Cristina Millán, José Altamirano, Vicente Arroyo, Joan Caballería, Ramón Bataller, Pere Ginès, Pau Sancho-Bru

**Affiliations:** 1 Institut d’Investigacions Biomèdiques August Pi i Sunyer (IDIBAPS), Barcelona, Spain; 2 Vall d’Hebrón Institut de Recerca (VHIR), Barcelona, Spain; 3 Centro de Investigación Biomédica en Red de Enfermedades Hepáticas y Digestivas (CIBERehd), Barcelona, Spain; 4 Liver Unit, Hospital Clínic, Faculty of Medicine, University of Barcelona, Barcelona, Spain; 5 Division of Gastroenterology and Hepatology, Departments of Medicine and Nutrition, University of North Carolina, Chapel Hill, North Carolina, United States of America; Institute of Hepatology, Foundation for Liver Research, UNITED KINGDOM

## Abstract

Chronic liver diseases are characterized by a sustained inflammatory response in which chemokines and chemokine-receptors orchestrate inflammatory cell recruitment. In this study we investigated the role of the chemokine receptor CCR6 in acute and chronic liver injury. In the absence of liver injury *Ccr6* ^*-/-*^ mice presented a higher number of hepatic macrophages and increased expression of pro-inflammatory cytokines and M1 markers *Tnf-α*, *Il6* and *Mcp1*. Inflammation and cell recruitment were increased after carbon tetrachloride-induced acute liver injury in *Ccr6* ^*-/-*^ mice. Moreover, chronic liver injury by carbon tetrachloride in *Ccr6* ^*-/-*^ mice was associated with enhanced inflammation and fibrosis, altered macrophage recruitment, enhanced CD4^+^ cells and a reduction in Th17 (CD4^+^IL17^+^) and mature dendritic (MHCII^+^CD11c^+^) cells recruitment. Clodronate depletion of macrophages in *Ccr6* ^*-/-*^ mice resulted in a reduction of hepatic pro-inflammatory and pro-fibrogenic markers in the absence and after liver injury. Finally, increased CCR6 hepatic expression in patients with alcoholic hepatitis was found to correlate with liver expression of CCL20 and severity of liver disease. In conclusion, CCR6 deficiency affects hepatic inflammatory cell recruitment resulting in the promotion of hepatic inflammation and fibrosis.

## Introduction

Alcoholic liver disease (ALD), non-alcoholic liver steatohepatitis (NASH) and viral infections represent some of the most common chronic injuries that promote liver inflammation, fibrosis and cirrhosis. The interplay between inflammatory damage and altered matrix deposition causes the slow accumulation of scar matrix, leading to progressive fibrosis and cirrhosis [1]. Cirrhosis is a severe clinical condition associated with bad prognosis, which can progress to hepatocellular carcinoma (HCC), one of the major causes of liver-related death worldwide [2]. There are currently no therapies available to treat cirrhosis, and despite recent studies having shown that it may be a reversible process, its resolution is still not achievable[1, 3]. In order to prevent the development of cirrhosis, it is important to further investigate the mechanisms leading to this severe condition. Alcoholic hepatitis (AH) is one of the most severe conditions in ALD and a cause of acute-on-chronic liver failure. It is associated with short term-mortality and characterized by hepatocellular damage and strong inflammatory response [4, 5].

Chemokines are small chemotactic molecules, which elicit their effects by binding and activating their transmembrane receptors. Several studies have shown that the deletion of specific chemokines or chemokine-receptors can affect liver pathophysiology favoring either promotion or inhibition of hepatic inflammation and fibrosis, thereby drawing attention to their use for therapeutic purposes [6, 7]. Moreover, studies in experimental models have revealed that chemokines and chemokine-receptors [8] play an important role in hepatic inflammation and fibrosis by orchestrating the inflammatory recruitment of different cell populations including macrophages, dendritic cells and T cells to the injured liver [9–11]. Several studies have also shown that chemokines and chemokine-receptors are differentially regulated in ALD suggesting that they may be important mediators in the progression of the disease [12–14]. Hepatic macrophages are well-known inflammatory cell populations involved in the pathogenesis of chronic liver diseases. They have been described to be important mediators of hepatic inflammation and fibrosis, participating in both progression and regression of fibrosis [15–17]. Therefore, macrophages have been recently been proposed as new potential targets to develop anti-fibrotic therapies [18].

In a recent study by our group we showed that chemokine CCL20 is a driver of hepatic inflammation and fibrosis in patients with AH, providing evidence that CCL20 mediates inflammatory cell recruitment upon liver injury by promoting hepatic infiltration of macrophages and neutrophils [19]. Chemokine receptor 6 (CCR6) is the only receptor described for the chemokine CCL20. CCR6 has been shown to be expressed in different inflammatory cells including CD4 T-cells and specifically in T-helper (Th)17 and T-regulatory (Treg) cells [20–23], dendritic cells [24, 25] and also in γδ T cells [26, 27]. Importantly, a recent study showed that CCR6-dependent accumulation of IL-17 producing γδ T cells restricts hepatic inflammation and fibrosis [28]. Nevertheless, the role of CCR6 during liver injury and how CCR6-dependent infiltrated cells mediate the progression of hepatic inflammation and fibrosis in response to liver injury is still largely unknown.

In this study we investigated the role of CCR6 in both acute and chronic liver injury. First, we showed that CCR6 deficiency affected hepatic inflammatory cell recruitment in both the absence and presence of liver damage. Moreover, CCR6 deficiency promoted exacerbated inflammation and fibrosis in experimental models of liver injury. Second, we provided evidences that the presence of both macrophages and CCR6-recruited cells is required for an adequate response to liver injury. Finally, in a cohort of patients with AH we showed the association between CCR6 hepatic expression and cirrhosis and its correlation with clinical scores of disease severity.

## Materials and Methods

### Ethics statement

All protocols were conformed to the ethical guidelines of the 1975 Declaration of Helsinki and were approved by the Ethics Committee of the Hospital Clinic of Barcelona. All the patients included in this study provided written and signed informed consent.

All animal procedures were approved by the Investigation and Ethics Committee of Animal Experimentation of the University of Barcelona and were conducted in accordance with the National Institutes of Health Guide for the Care and Use of Laboratory Animals. Euthanasia of animals used in this study was performed by overdose of a cocktail mix composed by an anaesthetic (ketamine) and an analgesic (xylazine).

### Experimental models of liver injury

Briefly, C57Bl/6 wild type (wt) and *Ccr6* knock out (*Ccr6* ^*-/-*^ *)* mice aged 8–12 weeks, were administered carbon tetrachloride (CCl4) to induce acute or chronic liver injury. Mononuclear inflammatory cells were isolated from the liver of mice chronically treated with CCl4, and were stained and analyzed by flow cytometry. The depletion of macrophages was performed by clodronate liposomes (clodronateliposomes.org, Amsterdam, The Netherlands) before injecting mice with vehicle (corn oil) or CCl4 to induce acute liver damage. The assessment of liver inflammation and fibrosis was performed by immunohistochemistry and Sirius red staining, respectively. All procedures are extensively described in S1 Text.

### Patients

*CCR6* hepatic gene expression was determined in 46 liver samples obtained by transjugular biopsy from patients with AH. The patients prospectively included in the study were admitted to the Liver Unit of the Hospital Clínic of Barcelona with clinical, analytical and histological features of AH from July 2009 to January 2012. The inclusion and exclusion criteria of AH have been previously described [19].

### Statistical analysis

Results of quantitative variables are expressed as mean ± standard error unless otherwise specified. Comparisons between groups were performed using the Student´s *t* test or the Mann-Whitney *U* test when appropriate. Correlations between variables were evaluated using Spearman’s *Rho* or Pearson’s *r*, when appropriate. Comparisons were performed by the log-rank test. All statistical analyses were performed using SPSS version 14.0 for Windows (SPSS Inc., Chicago, IL).

## Results

### *Ccr6* deficiency alters hepatic inflammatory recruitment in the absence of liver damage and after acute liver injury

**Fig 1:**
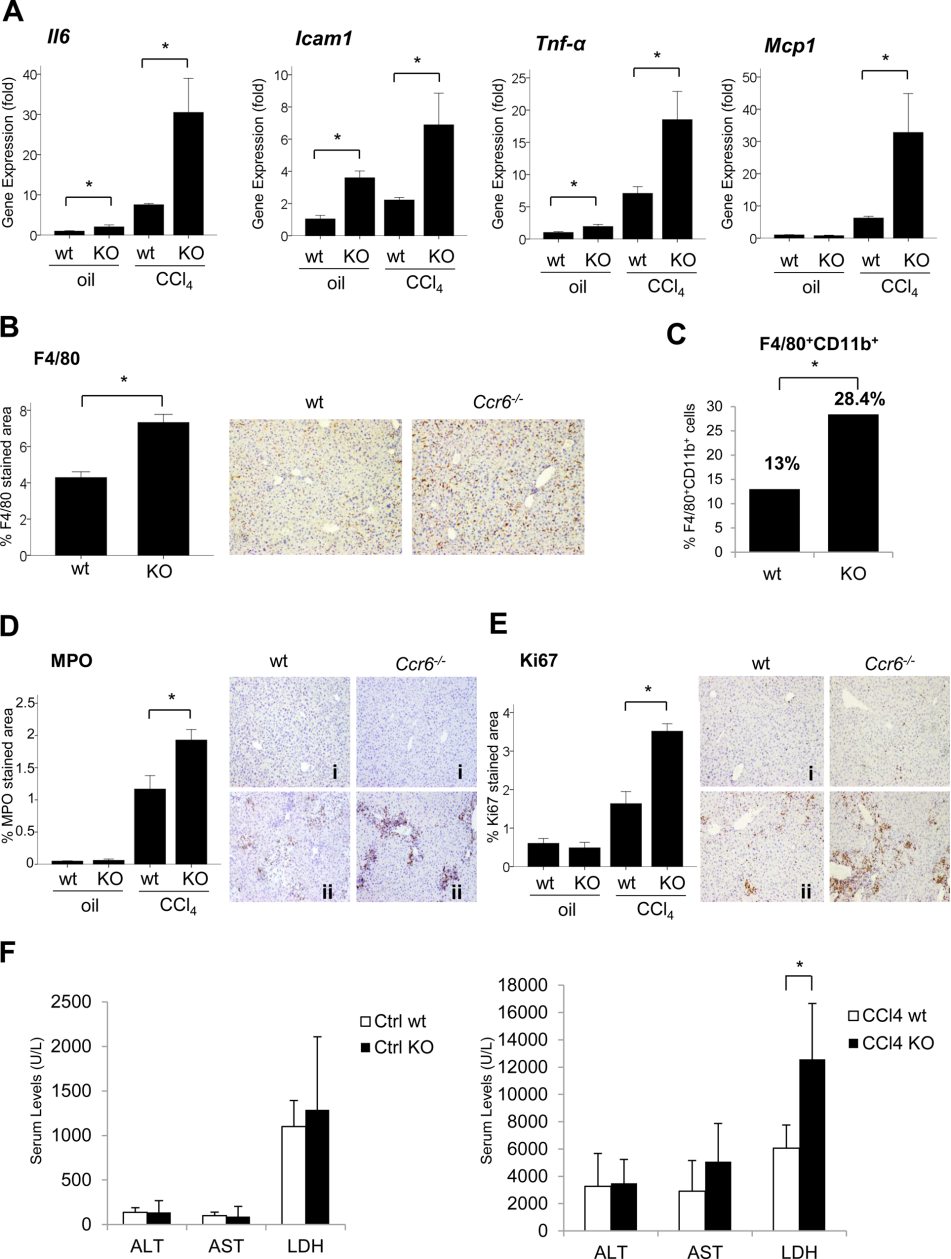
CCR6 in acute liver injury. (A) *Il6*, *Icam1*, *Tnf-α*, *Mcp1* hepatic gene expression in wt and *Ccr6* ^*-/-*^ (KO) mice in the absence (corn oil injected, n = 3 each group) and the presence of CCl4-induced acute liver injury (n = 4 each group) (*p<0.05 compared to wt). (B) Representative F4/80 immunostaining of liver sections of wt and *Ccr6* ^*-/-*^ (KO) mice (x200 magnification) in the absence of liver injury; F4/80 quantification of positive-stained areas in the absence of liver injury is shown in the graph (*p<0.05 compared to wt). (C) Percentage of macrophages (F4/80^+^CD11b^+^) in wt and *Ccr6* ^*-/-*^ mice (13±2.3% and 28.4±11.2% of total hepatic mononuclear cells respectively) in absence of liver injury. (D-E) Representative MPO and Ki67 immunostaining of liver sections of wt and *Ccr6* ^*-/-*^ (KO) mice (x200 magnification) in the absence (i) and presence (ii) of CCl4-induced acute liver injury; MPO and Ki67 quantification of positive-stained areas is shown in the graph (*p<0.05 compared to wt). (F) ALT, AST and LDH serum levels in wt and *Ccr6* ^*-/-*^ (KO) mice in the absence (ctrl group) (corn oil injected, n = 3 each group) and presence of CCl4-induced acute liver injury (n = 4 each group) (*p<0.05 with respect to wt).

In order to investigate the role of CCR6 in liver injury, we evaluated inflammatory cell recruitment in wt and *Ccr6* ^*-/-*^ mice in the absence and presence of liver injury. In the absence of damage, *Ccr6* ^*-/-*^ mice showed increased hepatic levels of well-known pro-inflammatory and macrophage-type1 (M1) markers such as *Il6*, *Icam1*, and *Tnf-α* (p<0.05) (Fig 1A). Moreover, we also detected an increased number of macrophages in the liver of *Ccr6* ^*-/-*^ mice compared to wt, as detected by F4/80 immunohistochemistry (p<0.05) (Fig 1B) and flow cytometry analyzing the F4/80^+^CD11b^+^ cell population (28.4±11.2% *Ccr6* ^*-/-*^ vs. 13±2.3% wt of total hepatic mononuclear cells) (p<0.05) (Fig 1C).

To evaluate the effects of *CCR6* during acute liver injury, mice were subjected to acute CCl4 treatment. In response to acute CCl4 administration, *Ccr6* ^*-/-*^ mice showed significantly increased hepatic levels of the pro-inflammatory molecules *Il6*, *Icam1*, *Tnf-α* and *Mcp1* as compared to wt (p<0.05) (Fig 1A). Importantly, we also observed a significant increase in neutrophil recruitment and increased inflammatory cell proliferation in *Ccr6* ^*-/-*^ mice subjected to acute liver damage as assessed by myeloperoxidase (MPO) and Ki67 immunohistochemistry respectively (p<0.05 for both) (Fig 1D and 1E). Finally, no changes in transaminases levels were observed in the absence of damage or after acute CCl4-induced liver injury. Moreover, CCl4 induced an increase in LDH serum levels as compared to wt (p<0.05) in *Ccr6* ^*-/-*^ mice (Fig 1F).

### *Ccr6* ^*-/-*^ mice show increased fibrosis upon chronic liver injury

**Fig 2:**
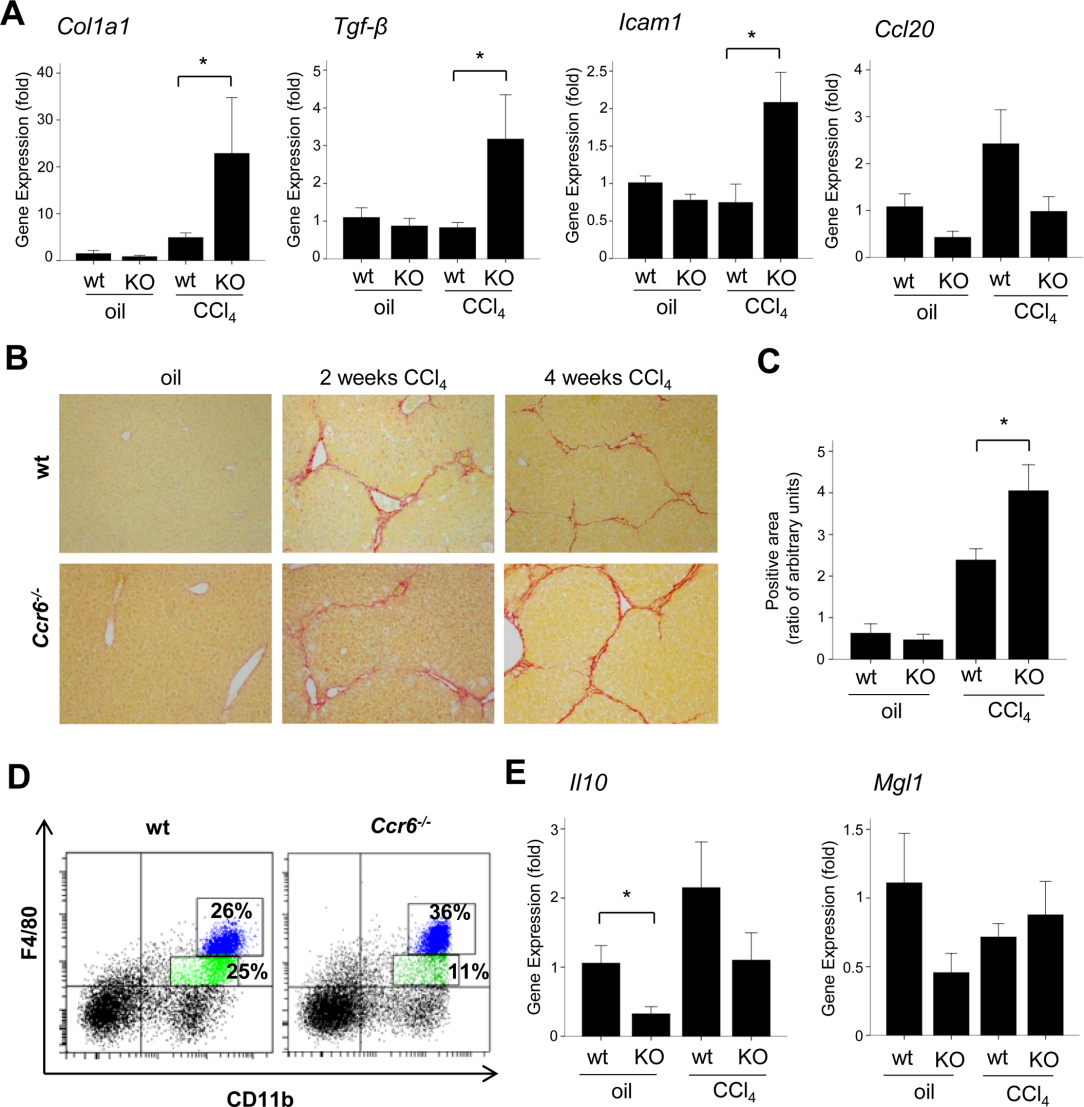
CCR6 in chronic liver injury. (A) *Col1a1*, *Tgf-β*, *Icam1*, *Ccl20* hepatic gene expression in wt (n = 8) and *Ccr6* ^*-/-*^ (KO) (n = 8) mice vehicle- (corn oil) or CCl4–treated for two weeks (see *S1 Text*) (*p<0.05 with respect to wt). (B) Representative images of Sirius Red staining in livers of wt and *Ccr6* ^*-/-*^ mice (x200 magnification) vehicle- (corn oil) or CCl4-treated for two and four weeks (see S1 Text). (C) Sirius Red quantification of positive-stained areas in vehicle- (corn oil) and four-week CCl4-treated wt and *Ccr6* ^*-/-*^ mice (*p<0.05 respect wt). (D) Representative FACS plots from livers of two weeks CCl4-treated mice showing F4/80low/CD11bmid/high monocytes in wt and *Ccr6* ^*-/-*^ mice (25±5.3% and 11±3.7% of total hepatic mononuclear cells, respectively) (p<0.005) and F4/80high/CD11bhigh freshly infiltrating monocyte-derived macrophages in wt and *Ccr6* ^*-/-*^ mice (26±6.1% and 36±8.8% of total hepatic mononuclear cells, respectively) (p = 0.08). (E) *Il10* and *Mgl1* hepatic gene expression in wt and *Ccr6* ^*-/-*^ two-week vehicle- (corn oil) or CCl4–treated mice (*p<0.05 respect wt).

To investigate the role of CCR6 during fibrogenesis in early phases of fibrosis and in a well-established fibrosis model, we assessed fibrosis in wt and *Ccr6* ^*-/-*^ animals by injecting mice with CCl4 during 2 and 4 weeks. After two weeks of CCl4-induced chronic liver injury, *Ccr6* ^*-/-*^ mice showed increased hepatic levels of pro-fibrogenic genes *Col1a1 and Tgf-*β and also increased *Icam1* compared to wt (p<0.05) (Fig 2A). Hepatic *Ccl20* expression showed no differences in *Ccr6* ^*-/-*^ compared to wt mice (Fig 2A). CCL20 protein expression was detected in hepatocytes after CCl4-induced chronic liver injury in wt and Ccr6^-/-^ mice (S1A Fig). No significant differences in the pro-inflammatory genes *Il6* and *Mcp1* were observed between the two groups in response to chronic liver injury (data not shown). The fibrosis level in *Ccr6* ^*-/-*^ mice was confirmed by Sirius Red staining. As shown in Fig 2B and 2C, we found a higher collagen deposition in the liver of *Ccr6* ^*-/-*^ mice after 2 and 4 weeks of chronic CCl4 treatment compared to wt (p<0.05).

### Inflammatory recruitment in *Ccr6* ^*-/-*^ mice in response to chronic liver injury

Since macrophages were found to be increased in *Ccr6* ^*-/-*^ mice in the absence of liver damage, we investigated the changes in macrophage population in *Ccr6* ^*-/-*^ and wt mice after chronic liver injury. Surprisingly, after induction of chronic liver injury we did not find any differences in the number of total macrophages (F4/80^+^Cd11b^+^ positive cells) in the liver of *Ccr6* ^*-/-*^ compared to wt (47±11.79% *Ccr6* ^*-/-*^ vs. 51±10.22% wt of total hepatic mononuclear cells) (Fig 2D) as assessed by flow cytometry. However, a more detailed analysis showed a reduced number of F4/80low/CD11bmid/high monocytes in *Ccr6* ^*-/-*^ mice compared to wt (11±3.7% *Ccr6* ^*-/-*^ vs. 25±5.3% wt of total hepatic mononuclear cells) (p<0.005) and a tendency to increase the infiltrating monocyte-derived F4/80high/CD11bhigh macrophages (36±8.8% *Ccr6* ^*-/-*^ vs. 26±6.1% wt of total hepatic mononuclear cells) (p = 0.08) which have been described to express Ly6C and to be associate with liver injury and inflammation [11]. These results suggest an altered inflammatory cell recruitment in *Ccr6* ^*-/-*^ mice with an increased proportion of infiltrated pro-inflammatory macrophages (Fig 2D). According to these results, in the absence of injury we detected a reduced hepatic expression of well-described anti-inflammatory macrophage-type 2 (M2) markers *Il10* (p<0.05) and *Mgl1* (p = 0.12) in *Ccr6* ^*-/-*^ mice. No significant differences were observed in *Il10 and Mgl1* expression in *Ccr6* ^*-/-*^ mice compared with wt after chronic treatment with CCl4 (Fig 2E).

**Fig 3:**
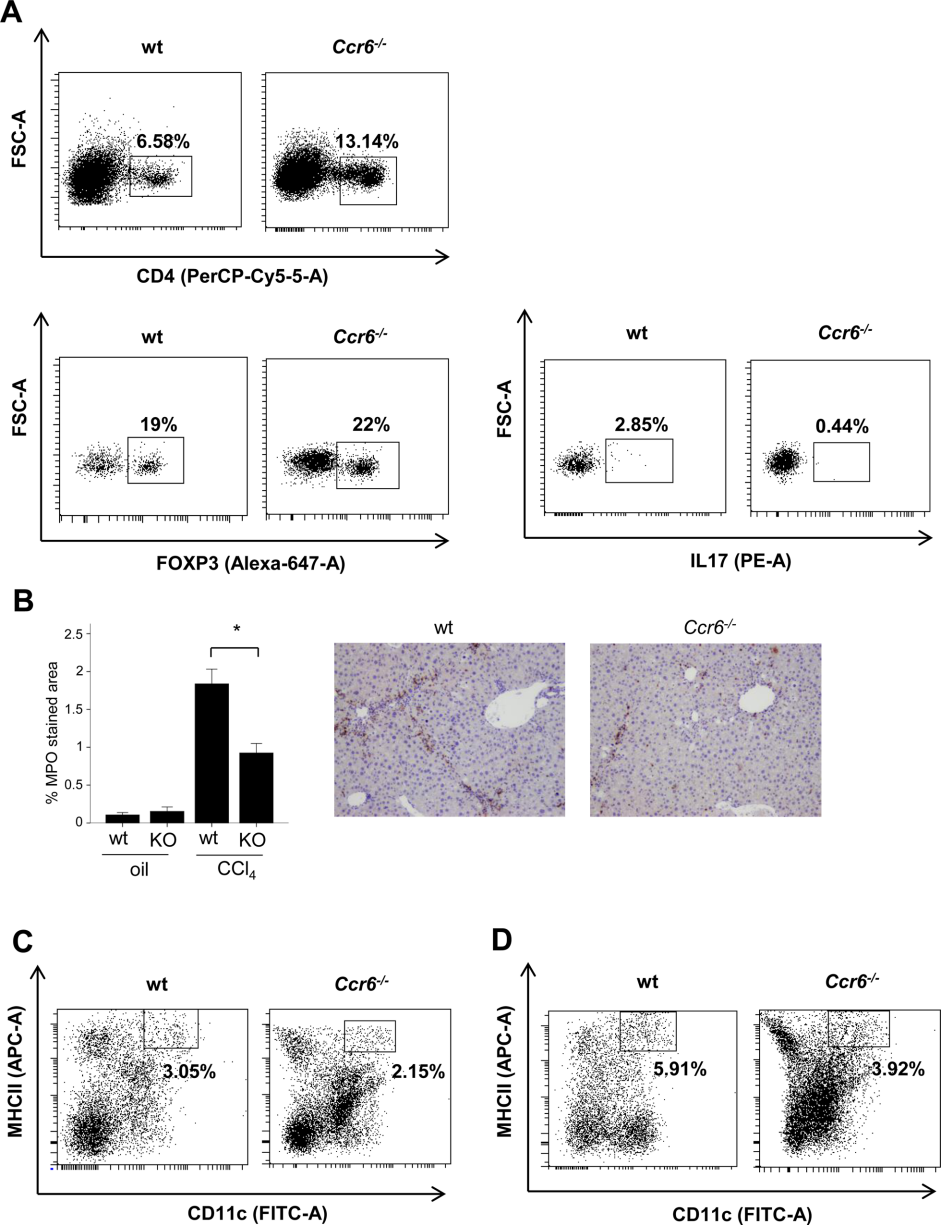
Effects of Ccr6 depletion on the hepatic inflammatory infiltrate. (A) Representative FACS plots of inflammatory cells isolated from two-week CCl4-treated mice livers showing percentage of CD4^+^T cells in wt and *Ccr6* ^*-/-*^ mice (5.56±2% in wt vs. 13.14±4.7% in *Ccr6* ^*-/-*^ of total hepatic mononuclear cells) (p<0.005), CD4^+^FOXP3^+^ (19±8.3% in wt vs. 22±5.5% in *Ccr6* ^*-/-*^ of CD4^+^T cells), CD4^+^IL17^+^ cells (2.85±1.5% in wt vs. 0.44±0.3% in *Ccr6* ^*-/-*^ of CD4^+^ cells) (p = 0.005). (B) Representative MPO immunostaining of liver sections of wt and *Ccr6* ^*-/-*^ (KO) mice treated for two weeks with CCl4 (x200 magnification); MPO quantification of positive-stained areas is shown in the graph (*p<0.05 compared to wt). (C) Representative FACS plots showing MHCII^+^CD11c^+^ cells in wt and *Ccr6* ^*-/-*^ mice in the absence of liver injury (3.05±0.5% in wt vs. 2.15±0.4% in *Ccr6* ^*-/-*^ of total hepatic mononuclear cells) (p<0.05). (D) after chronic CCl4-induced liver damage (5.91±1.5% in wt vs. 3.92±1.5% in *Ccr6* ^*-/-*^ of total hepatic mononuclear cells) (p<0.05).

In order to evaluate the effect of *Ccr6* deficiency, inflammatory cell recruitment was evaluated in wt and *Ccr6* ^*-/-*^ liver. After chronic CCl4 treatment, *Ccr6* ^*-/-*^ mice showed an increased number of total CD4^+^T cells (13.14±4.7% *Ccr6* ^*-/-*^ vs. 5.56±2% wt of total hepatic mononuclear cells) (p<0.005), no differences in the hepatic recruitment of CD4^+^FOXP3^+^ regulatory T cells (T-Reg) (22±5.5% *Ccr6* ^*-/-*^ vs. 19±8.3% wt of CD4^+^ cells), and a strong reduction in the number of CD4^+^IL17^+^ (Th-17) cells (0.44±0.3% *Ccr6* ^*-/-*^ vs. 2.85±1.5% wt of CD4^+^ cells) (p = 0.005) as compared to wt (Fig 3A) confirming that Ccr6 affects the hepatic T-cell recruitment [28]. To better define if chronic liver injury drives the recruitment of profibrotic Th2 cells, we assessed the hepatic levels of Th2 cytokines *ll4* and *Il13 as well as the* Th2 transcription factor *Gata3*. We did not find significant difference in *ll4 and Gata3* hepatic expression between Ccr6^-/-^ and wt mice, and no *Il13* expression was observed in the absence or presence of liver injury (S1B Fig). These results suggest that no Th2 compensatory mechanism might be involved in Ccr6^-/-^ mice phenotype. To investigate hepatic neutrophil recruitment after liver injury, we stained wt and *Ccr6* ^*-/-*^ liver samples with an MPO antibody. Interestingly, the hepatic number of MPO-positive cells was lower in *Ccr6* ^*-/-*^ mice compared to wt after chronic liver injury (p<0.05) (Fig 3B).

Finally, because dendritic cells have also been described to express CCR6 receptor, we investigated how CCR6 deficiency affected the hepatic recruitment of this cell population in response to chronic liver injury. Using flow cytometry analysis we observed a decreased number of MHCII^+^CD11c^+^ positive cells (mature dendritic cells) in *Ccr6* ^*-/-*^ mice compared to wt in the absence of liver injury (2.15±0.4% *Ccr6* ^*-/-*^ vs 3.05±0.5% *wt* of total hepatic mononuclear cells) (p<0.05) and after chronic CCl4-induced liver damage (3.92±1.5% *Ccr6* ^*-/-*^ vs 5.91±1.5% wt of total hepatic mononuclear cells) (p<0.05) (Fig 3C and 3D). No changes in the number of B220^+^CD11c^+^ positive cells (immature dendritic cells) were found in any of the groups (data not shown).

### Macrophage depletion critically reduces the liver expression of pro-inflammatory and pro-fibrogenic genes in *Ccr6* ^*-/-*^ mice

**Fig 4:**
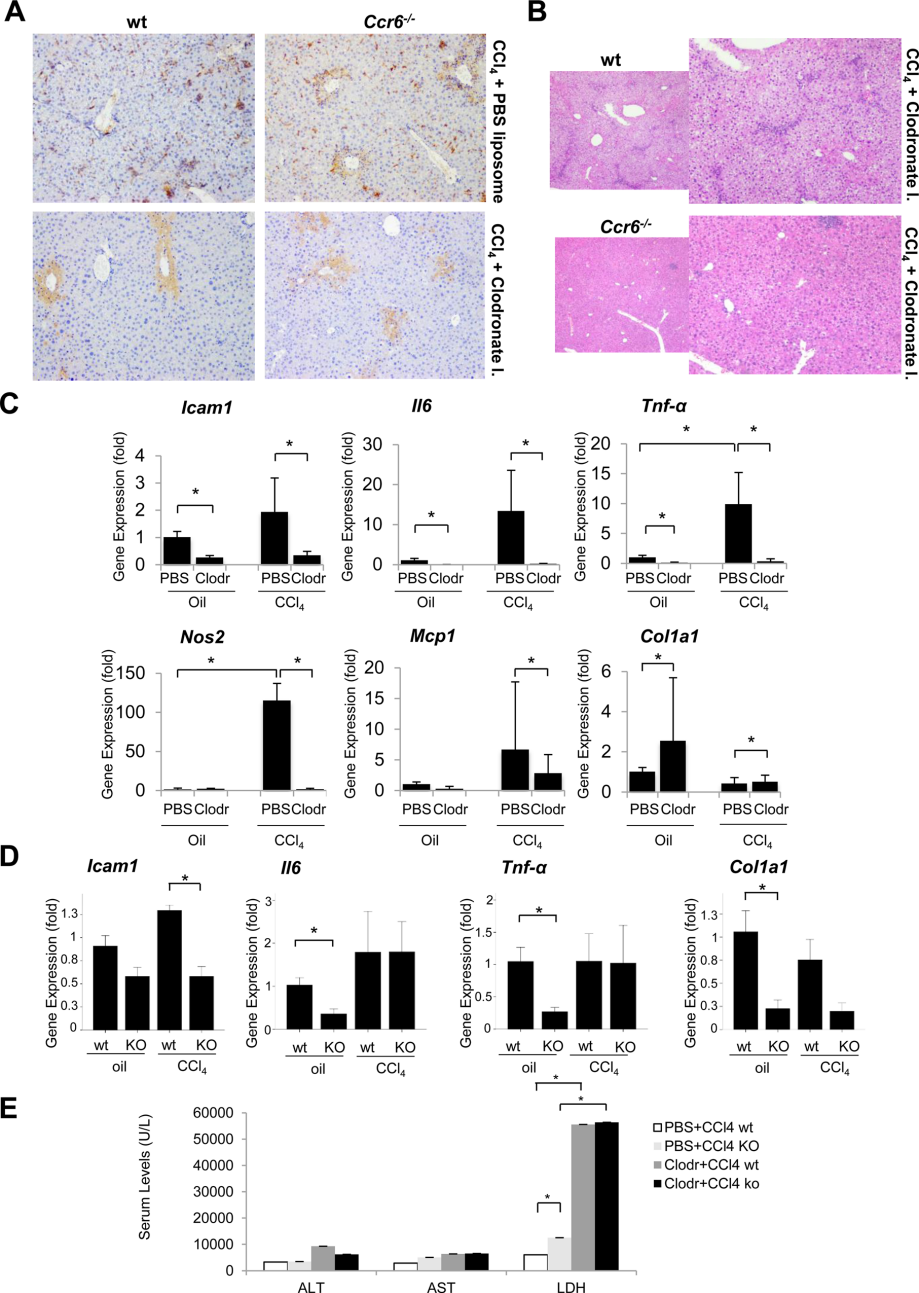
Effect of macrophage depletion in *Ccr6^-/-^* mice. (A) Representative F4/80 immunostaining of liver sections of wt and *Ccr6* ^*-/-*^ mice injected with PBS liposomes (vehicle) (n = 8) or clodronate liposomes (n = 13) followed by CCl4 acute liver injury (see *S1 Text*). (B) Representative H&E staining of liver sections (x100 magnification) of wt and *Ccr6* ^*-/-*^ mice injected with clodronate liposomes followed by CCl4 acute liver injury (see S*1 Text*). (C) *Icam1*, *Il6*, *Tnf-α*, *Nos2*, *Mcp1* and *Col1a1* hepatic gene expression in *Ccr6* ^*-/-*^ (KO) mice injected with PBS liposomes or clodronate liposomes in presence (1xCCl4–injected) or absence (vehicle, corn-oil injected) of CCl4-induced acute liver damage (see *S1 Text*) (*p<0.05). (D) *Icam1*, *Il6*, *Tnf-α* and *Col1a1* hepatic gene expression in wt and *Ccr6* ^*-/-*^ (KO) mice injected with clodronate liposomes and vehicle- (corn oil) or 1xCCl4 (*p<0.05 with respect to wt). (E) ALT, AST and LDH serum levels in wt and *Ccr6* ^*-/-*^ (KO) mice in the presence (PBS liposomes injected) and absence (clodronate liposomes injected) of macrophages in animals with CCl4-induced acute liver injury (*p<0.05).

On finding an increased number of macrophages in the uninjured livers of *Ccr6* ^*-/-*^ mice, we investigated whether macrophages might be responsible for the increased levels of inflammatory mediators and the exacerbated inflammatory response observed in *Ccr6* ^*-/-*^ mice. Macrophages were depleted by injecting wt and *Ccr6* ^*-/-*^ mice intraperitoneally with clodronate- or PBS-liposomes. The effect on hepatic inflammation and fibrosis was assessed after CCl4-induced acute liver damage. The hepatic depletion of macrophages in the absence (data not shown) and the presence of liver injury were assessed by F4/80 immunohistochemistry. Effects of clodronate treatment were assessed by hematoxylin and eosin and F4/80 staining in liver sections of Ccr6-/- and wt mice injected with vehicle (PBS liposomes) or clodronate liposomes followed by one single intraperitoneal injection of CCl4. (Fig 4A and 4B). Quantification of F4/80 positive cells in wt mice (24.36±2.74 F4/80^+^ cells/field in PBS liposomes plus CCl4 vs 0.25±0.26 F4/80^+^ cells/field in clodronate liposomes plus CCl4) and in *Ccr6* ^*-/-*^ mice (25.44±3.98 F4/80^+^ cells/field in PBS liposomes plus CCl4 vs 0.08±0.13 F4/80^+^ cells/field in clodronate liposomes plus CCl4) confirmed the depletion of macrophages after clodronate treatment (Fig 4A).

As shown in Fig 1A, *Ccr6* ^*-/-*^ mice presented increased levels of pro-inflammatory and pro-fibrogenic genes. Interestingly, the depletion of macrophages by clodronate liposomes in *Ccr6* ^*-/-*^ mice in the absence or after CCl4-induced acute liver damage induced a strong reduction of *Icam1*, *Il6*, *Tnf-α*, *Nos2*, *Mcp1* and *Col1a1* compared to control liposomes injected mice (Fig 4C). Although clodronate liposome also induced a reduction of hepatic inflammatory genes *Il6*, *Tnf-α* and *Mcp1* in wt mice (S2A Fig), macrophage depletion in *Ccr6* ^*-/-*^ mice lowered inflammatory gene expression to that of wt mice or lower (Fig 4D). After acute CCl4 treatment in macrophage-depleted animals, we observed a strong increase in LDH levels in both wt and in *Ccr6* ^*-/-*^ mice, but no significant differences were found between the two groups. No changes in ALT and AST levels were detected between groups in the presence or the absence of macrophages (Fig 4E) suggesting that hepatocellular damage may not be affected. The response to liver injury in mice after macrophage depletion was investigated only in acute liver injury setting since *Ccr6* ^*-/-*^ mice treated with clodronate liposomes were extremely sensitive to CCl4 treatment, which induced at 24 hours a 62% mortality compared to the 12.5% observed in macrophage-depleted and CCl4-injected wt mice. In order to understand the potential causes of an increased mortality in *Ccr6* ^*-/-*^ mice we performed a time course mortality study to evaluate whether differences observed in *Ccr6* ^*-/-*^ mice were associated with changes in liver injury. No mortality was observed at 4 hours after clodronate plus CCl4 injection. Surprisingly, although mortality was higher in *Ccr6* ^*-/-*^ mice at 8 (0% wt vs. 25% *Ccr6* ^*-/-*^ *mice*) and 12 hours (25% wt vs. 40% *Ccr6* ^*-/-*^ *mice*) transaminase levels were not different between Ccr6^-/-^ and wt animals. Moreover, gene expression levels of Treg (*Foxp3*), proliferation (*Cyclin D1*), pro-inflammatory (*Icam1*, *Il6*), pro-apoptotic (*Bax*), anti-apoptotic (*Bcl2*) and pro-fibrogenic (*Tgf-β*) genes at 4, 8 and 12 hours did not change between Ccr6^-/-^ and wt in macrophages-depleted surviving mice after single CCl4 injection (S3 Fig).

### Chemokine receptor CCR6 expression correlates with clinical features of alcoholic hepatitis

**Fig 5:**
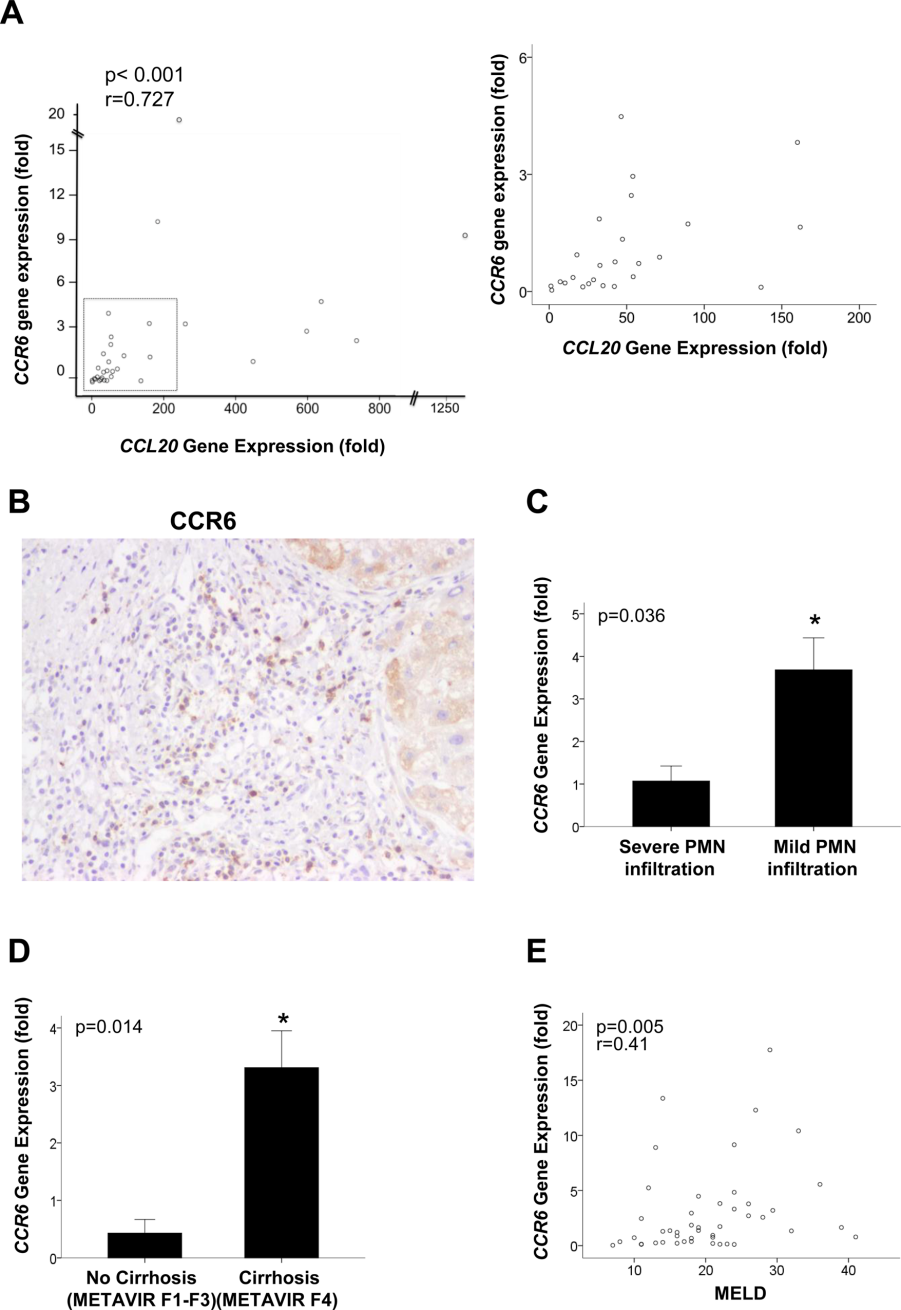
*CCR6* hepatic expression and correlation with clinical features of AH. (A) Correlation between *CCR6* and *CCL20* hepatic gene expression in patients with AH (n = 46) (p<0.001). (B) Representative image of CCR6 staining in a liver sample from a patient with AH (magnification x200). (C) Association between *CCR6* hepatic gene expression and hepatic PMN infiltration (p = 0.036). (D) Correlation between *CCR6* hepatic expression and the METAVIR prognostic score. (E) Correlation between *CCR6* hepatic gene expression and the MELD score in patients with AH (n = 46) (p<0.005).

Since CCR6 expression was found to regulate the liver inflammatory response to injury, we evaluated the expression of CCR6 in AH, a disease with an important inflammatory response and increased expression of CCL20. CCR6 expression was evaluated in a cohort of 46 well-characterized patients with AH with already reported levels of CCL20 [19]. Although we did not observe a statistically significant increase in *CCR6* hepatic expression in patients with AH (S4A Fig) compared to normal liver, we found that the expression of *CCR6* correlated with the hepatic expression of *CCL20* (Fig 5A), the only chemokine that reportedly binds and activates this receptor (p<0.001). In addition, we further investigated the protein expression of CCR6 in AH patients liver tissue. Interestingly, we found that CCR6 was mainly detected in inflammatory cells, especially in mature lymphocytes, but also it was found to be expressed in some hepatocytes (Fig 5B). However, as observed by a pathologist, not all lymphocytes were positive for CCR6, suggesting that probably, only a specific subset of these cells express CCR6 in this specific clinical condition.

Surprisingly, we found that *CCR6* hepatic expression was inversely correlated with the amount of infiltrated polymorphonuclear cells (PMN) in patients with AH, showing higher levels of CCR6 expression in patients with mild PMN infiltration with respect to those with severe PMN infiltration (Fig 5C) (p = 0.036). Moreover, in patients with AH, *CCR6* hepatic expression positively correlated with important prognostic scores such as METAVIR (p = 0.014) (Fig 5D), MELD (Model for End-stage Liver Disease) (p = 0.005) (Fig 5E), and Maddrey’s (p = 0.002) and ABIC (p = 0.043) scores (S4B and S4C Fig).

## Discussion

Inflammatory cell homeostasis and recruitment are essential for a well-controlled tissue inflammatory response. In this study we investigated the role of the CCR6 receptor in the hepatic homeostasis and recruitment of inflammatory cell populations and in the regulation of liver injury. Using *Ccr6* deficient mice, we showed that CCR6 deficiency had an important impact on inflammatory cell recruitment causing exacerbated inflammation and fibrosis. Moreover, in AH, a disease characterized by a high expression of CCL20, the only ligand of CCR6 described, CCR6 expression correlates with disease severity. Our results reveal CCR6 as an important cytokine-receptor in the regulation of the inflammatory cell recruitment and the modulation of liver damage.

Chemokines and chemokine-receptors have been described to play an active role in liver diseases by modulating hepatic inflammatory recruitment [11, 19, 28–30] and have been proposed as potential therapeutic targets [4, 6, 31, 32]. In a recent study we showed that CCL20 is a pleiotropic chemokine highly expressed in AH and mediates liver injury, inflammation and fibrosis [19]. Moreover, recent studies have linked CCL20 expression with CCR6-dependent recruitment of inflammatory cells, which modulate liver response to injury and liver regeneration [28, 33]. We have now assessed the expression and the role of CCR6 in the context of liver injury to understand the weight of CCR6-dependent recruitment of inflammatory cells in liver disease.

Frequently, strategies to inhibit a particular chemokine and chemokine-receptor pathway are not fully effective due to their high promiscuity. To overcome this limitation, in this study we used *Ccr6* knockout mice in order to ensure complete blockage of CCR6-dependent recruitment. Although our system does not prevent inflammatory cell population to be recruited through other receptors, our results indicate that CCR6 has an effect on inflammatory cell recruitment in the healthy liver and during liver injury. This is important since changes in inflammatory cell populations and their phenotype may be of key importance for liver response to injury. Interestingly, we observed that the hepatic Ccr6-dependent inflammatory recruitment affects several cell populations including neutrophils, T cells, dendritic cells and macrophages. Moreover, as detected by Ki67 immunohistochemistry, we also observed an increased proliferation of inflammatory cells in *Ccr6* ^*-/-*^ mice compared to wt animals, confirming exacerbated inflammation in the absence of Ccr6.

T lymphocytes and particularly CD4^+^T cells (T helper) have been described to be involved in the pathogenesis of chronic liver diseases affecting hepatocellular damage [6]. We observed that the deficiency of the CCR6 receptor has an important effect on the populations of lymphocytes recruited in the liver after both acute and chronic liver injury. CCR6 deficiency does not promote liver hepatocellular injury, however, LDH levels are increase after CCl4 treatment suggesting a potential effect on extrahepatic organs. We have shown that CCR6 deficiency not only mediates an increase in the recruitment of CD4^+^ positive cells but also determines a reduction of IL17^+^ positive cells, remarking that CCR6 participates in the recruitment of T helper cells in the liver in response to liver injury. Two recent studies provided important information on the mechanisms involved in the role of the CCL20-CCR6 axis in liver injury showing that the recruitment of γδT cells restricted liver injury and promoted liver regeneration [28, 33]. The adoptive transfer of wt γδT cells into Ccr6 deficient mice reduced hepatic inflammation and fibrosis in chronic injury, and the recruitment of γδT cells increased the production of IL17, which has been shown to promote hepatocyte proliferation and regeneration in partial hepatectomy [28, 33]. Our findings are in accordance with these previous studies by showing an association between CCR6-dependent inflammatory recruitment and the progression of liver inflammation and fibrosis. However, an important finding of our study is the observation that macrophages are differentially recruited in *Ccr6* deficient and *wt* mice. Furthermore, the phenotype of macrophages in healthy and injured liver of *Ccr6* deficient mice is altered and adopts a more inflammatory M1 profile.

Macrophages are a heterogenic population in the liver and important players in both acute and chronic liver injury. A key feature of macrophages is their plasticity and the acquisition of a M1 pro-inflammatory and M2 anti-inflammatory phenotype in response to inflammatory mediators, which have been shown to play an important role in fibrosis progression and resolution [17]. In this study we showed that the number of macrophages in non-injured *Ccr6* ^*-/-*^ mice is increased, and that the liver showed an increased expression of M1 markers *Tnf-α*, *Il6* and *Mcp1*. Moreover, although the number of macrophages did not vary between wt and *Ccr6* deficient mice after liver injury, *Ccr6* ^*-/-*^ mice presented an increased expression of hepatic M1 cytokines and an increased number of hepatic freshly infiltrating monocyte-derived macrophages as assessed by qPCR and flow cytometry. Whether, macrophages from *Ccr6* ^*-/-*^ mice show a different phenotype or a different response to injury as compared to those from wt animals is not addressed in this study and will deserve further investigation. We also observed a decreased number of MHCII+CD11c+ positive cells (dendritic cells) in Ccr6^-/-^ mice compared to wt in the absence and after liver injury. Since CD11c might be also present in liver macrophages under inflammatory conditions [34], in our study we have used a combination of CD11c with MHCII, which are both highly expressed in classical/mature dendritic cells [35, 36] to better discern mature dendritic cells and macrophages.

To investigate if the increase in macrophage number is responsible for the exacerbated inflammatory response and increased fibrosis observed in *Ccr6* ^*-/-*^ mice, we depleted the macrophages in healthy and injured mice. As expected, the depletion of macrophages in *Ccr6* ^*-/-*^ mice induced a decrease in the expression of inflammatory mediators to levels comparable to wt mice or even lower, suggesting that the unbalanced macrophage number and phenotype contributed to the altered hepatic inflammatory state in *Ccr6* ^*-/-*^. Surprisingly, *Ccr6* ^*-/-*^ mice depleted of macrophages were extremely susceptible to liver injury, which precluded us performing a chronic liver injury model. However, the analysis of macrophage-depleted *Ccr6* ^*-/-*^ mice at early time points after CCl4 administration, showed no effect on the extent of liver injury or inflammatory response as assessed by transaminase levels and pro-inflammatory cytokines expression. Whether macrophages in combination with CCR6-recruted cells have a protective effect to liver injury or promote a regenerative stimulus deserves further investigations.

Due to the overexpression of chemokine CCL20 in patients with AH, we also checked the hepatic expression of the receptor CCR6 in the same cohort of patients. Although we did not find significantly increased levels of CCR6 in the liver of these patients, as expected, CCR6 hepatic expression positively correlated with CCL20 hepatic expression. Interestingly, CCR6 hepatic expression was observed in inflammatory cells within the fibrous septa and some hepatocytes. Moreover, its expression correlated with important clinical scores defining the severity of the disease, suggesting a possible role of the CCL20/CCR6 axis in the pathogenesis of AH.

This study, together with previous data investigating the role of CCL20 in liver disease, identifies the CCL20/CCR6 pathway as an important player in liver disease. While CCL20 expression exerts an increase in liver injury and inflammation, here we show that the lack of CCR6 exacerbates the inflammatory cell response. This apparent opposite effects may be due to several reasons: the fact that CCL20 may be a ligand to other non-identified receptors, an inefficient signaling of CCL20 in CCR6 deficient animals, an altered recruitment of CCR6 inflammatory cells, or the alteration of the phenotype of inflammatory cells lacking the CCR6 receptor. Further studies will have to elucidate these open questions regarding the mechanisms driving the combined effects of CCL20 and CCR6 in the context of liver injury.

Here we show that deficiency of the CCR6 receptor mediates an alteration in inflammatory cell recruitment, which results in a worsened inflammatory and fibrogenic liver response to injury. Moreover, we highlight the prominent role of macrophages in this exacerbated response. Altogether, these results suggest that CCR6 deficiency exacerbates liver injury by increasing macrophage recruitment and promoting an inflammatory phenotype. Defining the role of chemokines and chemokine-receptors such as CCL20 and CCR6 in liver injury is of paramount importance to better understand liver disease and to develop new targeted therapeutic strategies.

## Supporting Information

S1 Checklist NC3Rs ARRIVE Guidelines Checklist 2014.). (DOCX)

S1 Fig CCL20 expression and Th2 cell recruitment in wt and Ccr6^-/-^ mice after two weeks of CCl_4_ treatment. (A) CCL20 immunostaining in wt and Ccr6^-/-^ mice treated with CCl4. (B) *Il4* and *Gata3* hepatic gene expression in wt and Ccr6^-/-^ mice treated chronically with vehicle (corn oil) or CCl4 (*p<0.05, ** p< 0.001).
(TIF)

S2 Fig Effect of macrophage depletion in wt mice. (A) *Icam1*, *Il6*, *Tnf-α*, *Nos2*, *Mcp1* and *Col1a1* hepatic gene expression in wt mice injected with PBS liposomes or clodronate liposomes in presence (1xCCl4–injected) or absence (vehicle, corn-oil injected) of CCl4-induced acute liver damage (see *S1 Text*) (*p<0.05).
(TIF)

S3 Fig Time course mortality study in wt and Ccr6^-/-^ macrophage depleted mice. Hepatic gene expression and ALT, AST and LDH serum levels in wt and Ccr6^-/-^ treated mice with clodronate liposome and single CCl4 injection at (A) 4 hours (n = 4 each group). (B) 8 hours (n = 4 each group) and (C) 12 hours (n = 4 wt mice, n = 5 Ccr6^-/-^ mice) post CCl4 injection (*p<0.05).
(TIF)

S4 Fig *CCR6* correlations with clinical scores of AH. (A) *CCR6* hepatic gene expression in patients with AH (n = 46). (B) Correlation between *CCR6* hepatic gene expression and Maddrey’s modified discriminant function in patients with AH (n = 46) (p = 0.002); (C) Correlation between *CCR6* hepatic gene expression and the ABIC score in patients with AH (n = 46) (p = 0.043).
(TIF)

S1 Text Supplementary Materials. (DOC)
