# Supplementary material for: Chemokine Receptor Ccr6 Deficiency Alters Hepatic Inflammatory Cell Recruitment and Promotes Liver Inflammation and Fibrosis
Source: PLoS One. 2015 Dec 21;10(12):e0145147. doi: 10.1371/journal.pone.0145147 (PMC4687007; doi:10.1371/journal.pone.0145147)

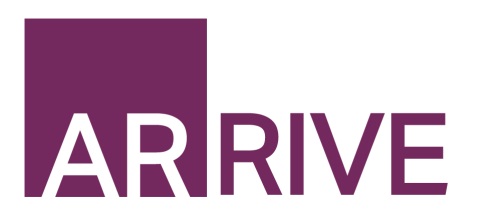


The ARRIVE Guidelines Checklist

Animal Research: Reporting In Vivo Experiments

Carol Kilkenny^1^, William J Browne^2^, Innes C Cuthill^3^, Michael Emerson^4^ and Douglas G Altman^5^

*^1^The National Centre for the Replacement, Refinement and Reduction of Animals in Research, London, UK, ^2^School of Veterinary Science, University of Bristol, Bristol, UK, ^3^School of Biological Sciences, University of Bristol, Bristol, UK, ^4^National Heart and Lung Institute, Imperial College London, UK, ^5^Centre for Statistics in Medicine, University of Oxford, Oxford, UK.*

|  | | ITEM | RECOMMENDATION | Section/ Paragraph |
| --- | --- | --- | --- | --- |
| 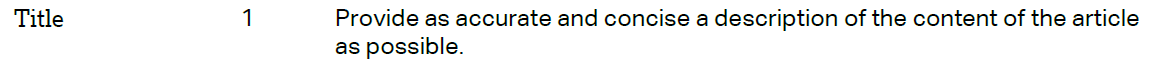 | | | Page 1 |  |
| 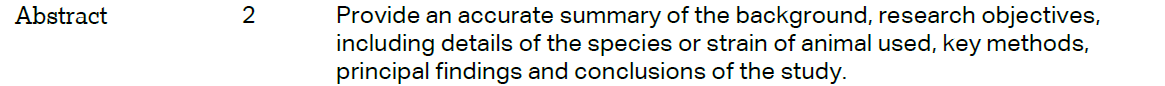 | | | Page 3 |  |
| INTRODUCTION | | |  |  |
| 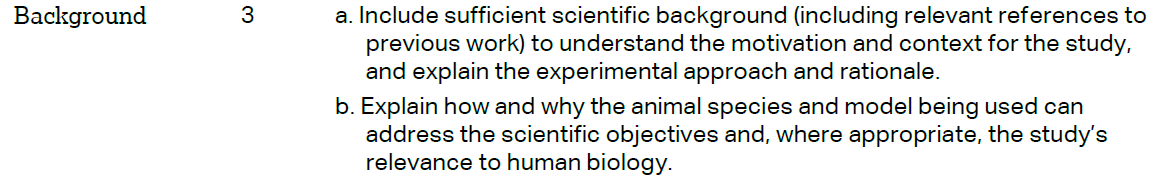 | | | Pages 4-5 |  |
| 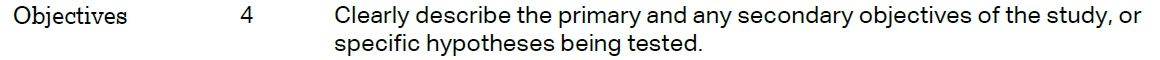 | | | Page 5, paragraph 3 |  |
| METHODS | | |  |  |
| 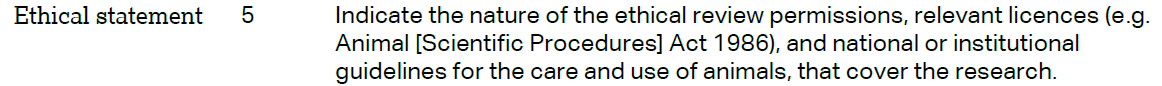 | | | Page 6, paragraph 2 |  |
| 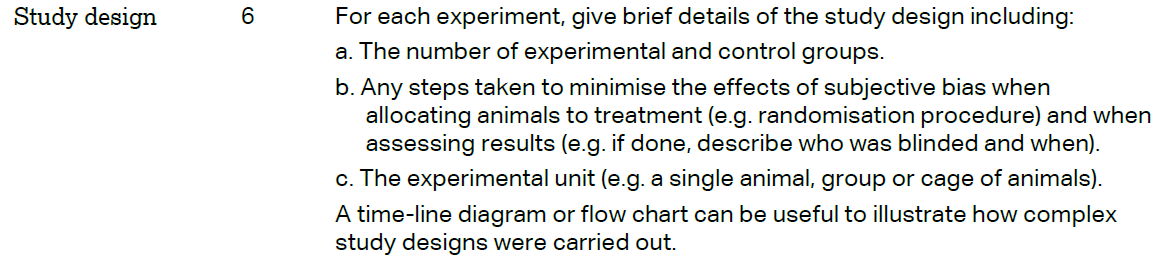 | | | Page 6, paragraph 3, Figure Legends (pages 22-24) and Supplementary Materials (Pages 1-2) |  |
| 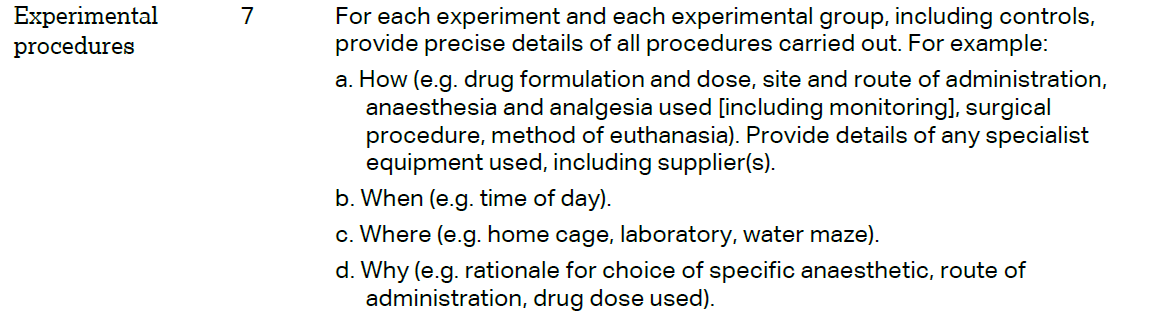 | | | Page 6, paragraph 3 and Supplementary Materials (Pages 1-2) |  |
| 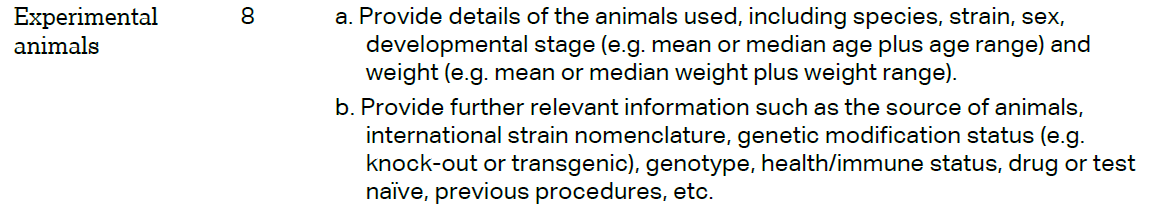 | | | Page 6, paragraph 3 and Supplementary Materials (Pages 1-2) |  |

The ARRIVE guidelines. Originally published in *PLoS Biology*, June 2010^1^

| 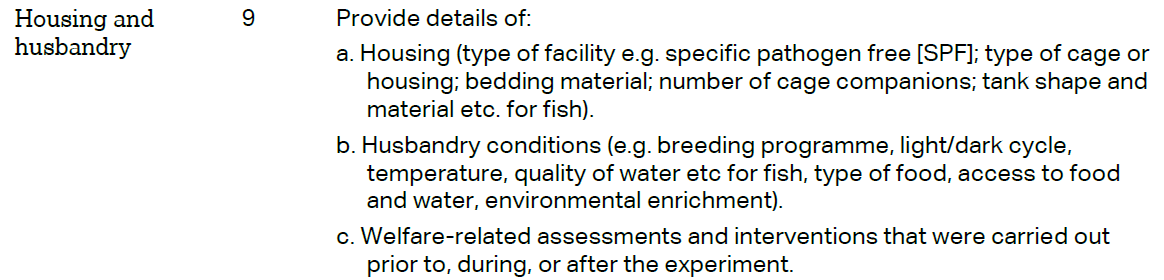 | Page 6, paragraph 3, and Supplementary Material (Pages 1-2) | |
| --- | --- | --- |
| 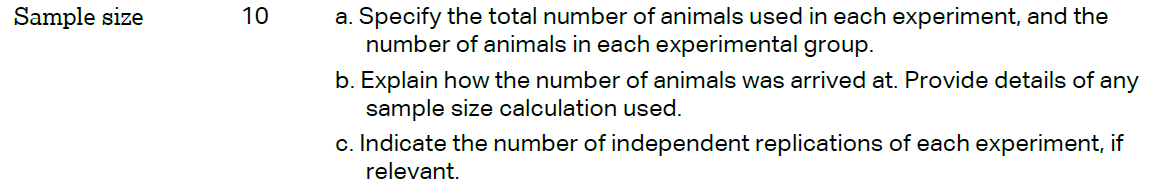 | Figure Legends 1-4 (Pages 22-23) | |
| 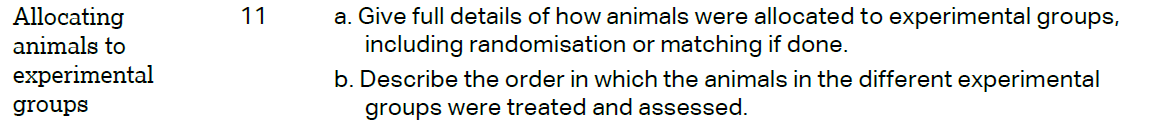 | Figure Legends 1-4 (Pages 22-23) | |
| 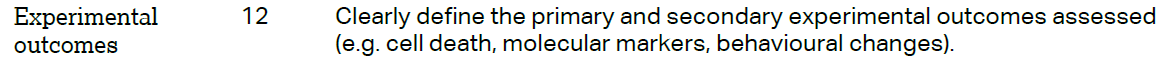 | Figure Legends 1-4 (Pages 22-23) and Supplementary Material (Pages 1-2) | |
| 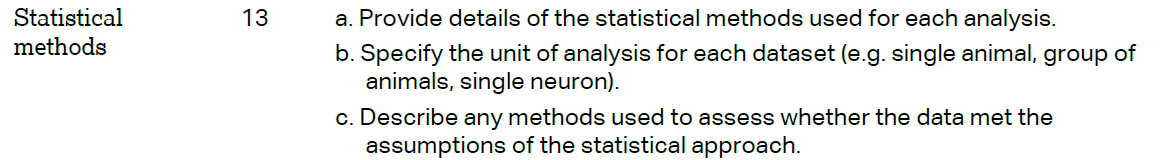 | Page 7 | |
| RESULTS |  | |
| 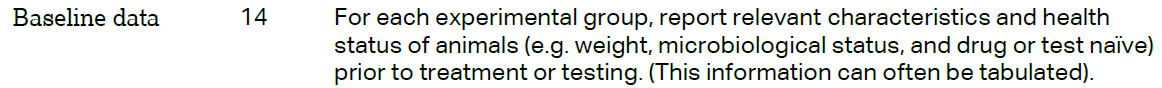 | Pages 8-11 | |
| 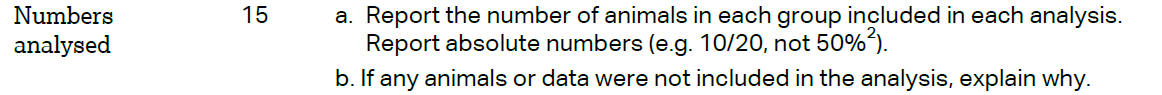 | Pages 8-11 and Figure Legends 1-4 (Pages 22-23) | |
| 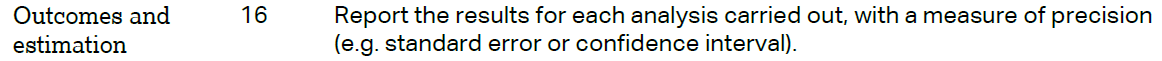 | Pages 8-11 | |
| 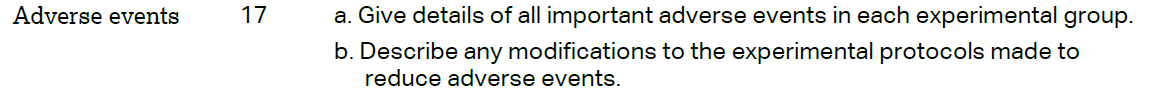 | Pages 8-11 | |
| DISCUSSION |  | |
| 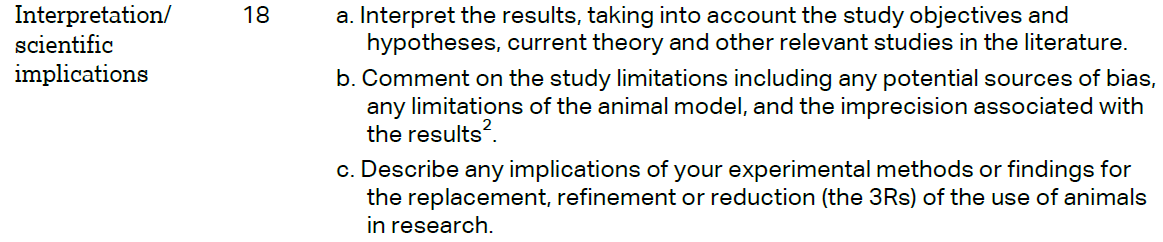 | Pages 13-16 | |
| 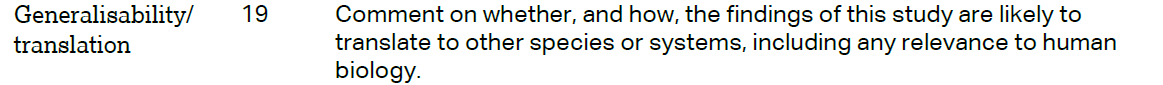 | Pages 13-16 | |
| 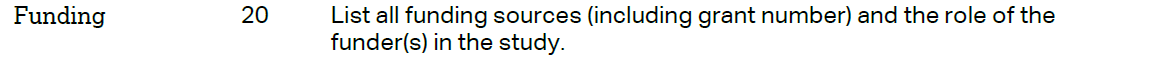 | | Online Submission |


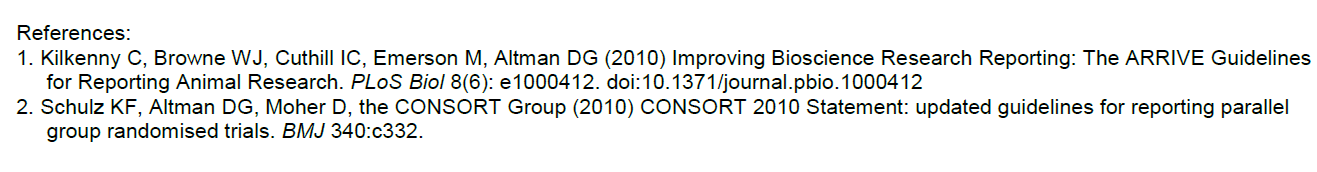

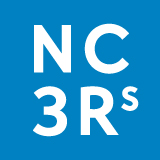

Supplement: S1 Checklist — (DOCX) [file pone.0145147.s001.docx]
