# Supplementary figures and images for: Chemokine Receptor Ccr6 Deficiency Alters Hepatic Inflammatory Cell Recruitment and Promotes Liver Inflammation and Fibrosis
Source: PLoS One. 2015 Dec 21;10(12):e0145147. doi: 10.1371/journal.pone.0145147 (PMC4687007; doi:10.1371/journal.pone.0145147)

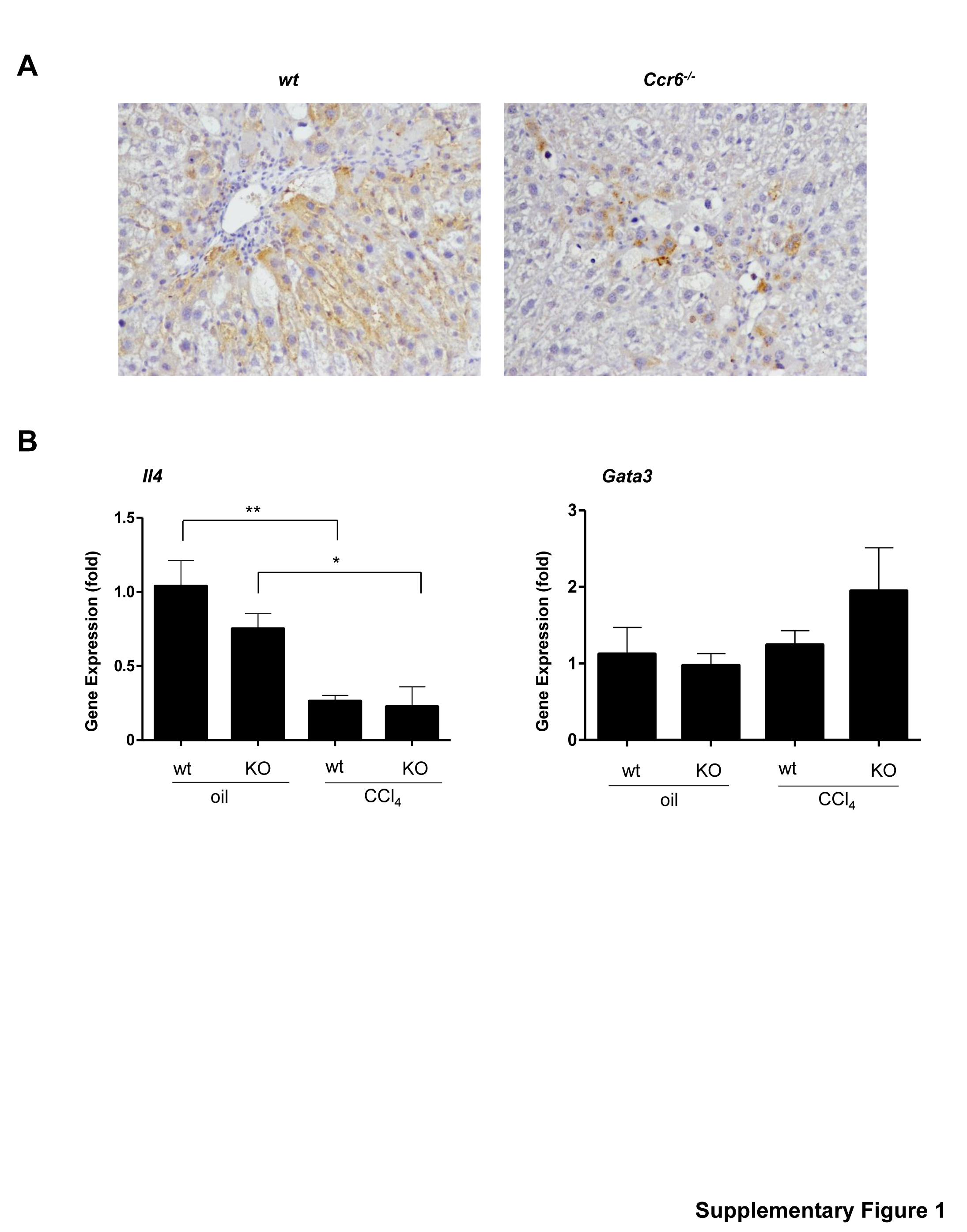

Supplement: S1 Fig — (A) CCL20 immunostaining in wt and Ccr6-/- mice treated with CCl4. (B) Il4 and Gata3 hepatic gene expression in wt and Ccr6-/- mice treated chronically with vehicle (corn oil) or CCl4 (*p<0.05, ** p< 0.001). (TIF) [file pone.0145147.s002.tif]

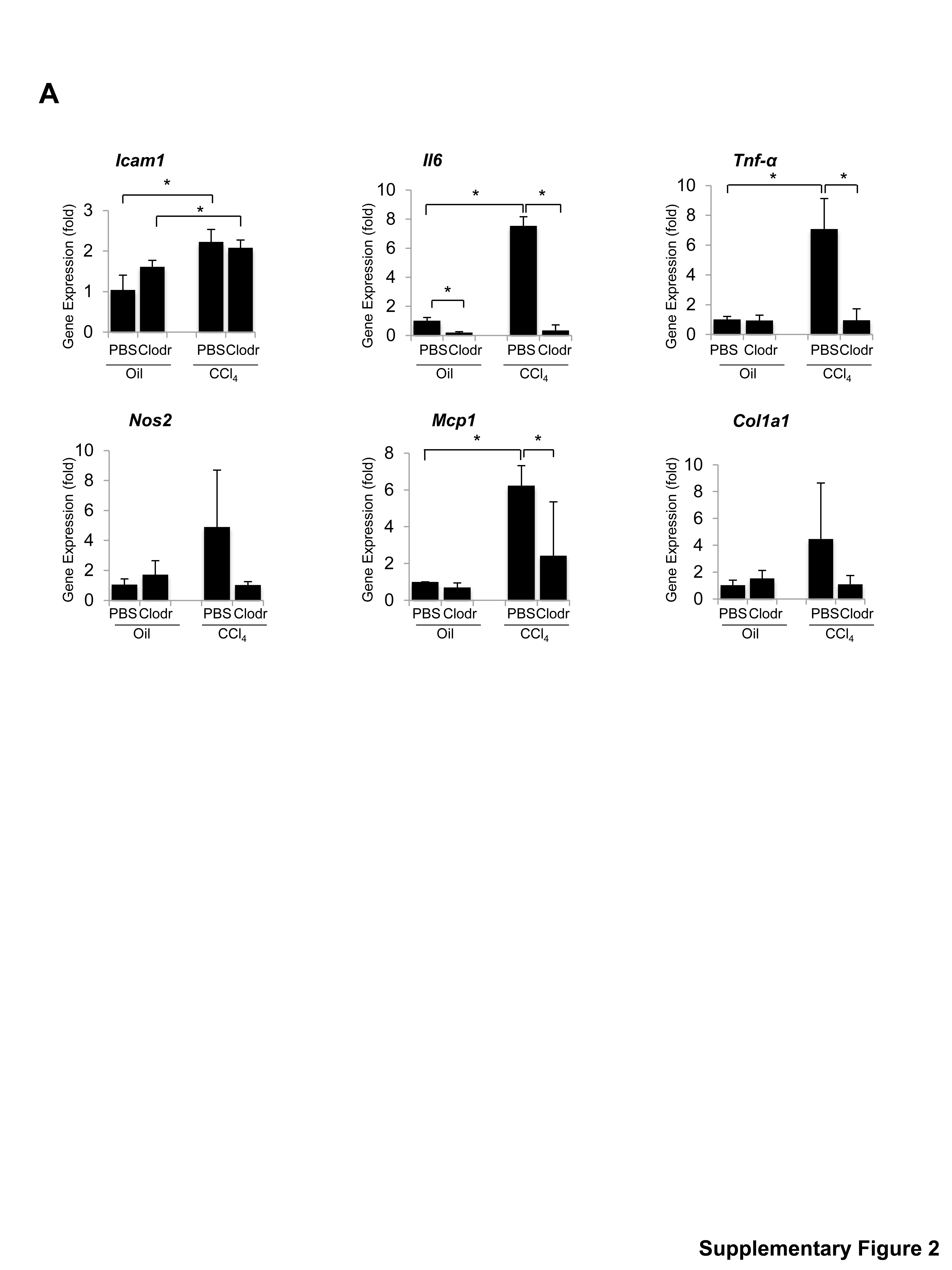

Supplement: S2 Fig — (A) Icam1, Il6, Tnf-α, Nos2, Mcp1 and Col1a1 hepatic gene expression in wt mice injected with PBS liposomes or clodronate liposomes in presence (1xCCl4–injected) or absence (vehicle, corn-oil injected) of CCl4-induced acute liver damage (see S1 Text) (*p<0.05). (TIF) [file pone.0145147.s003.tif]

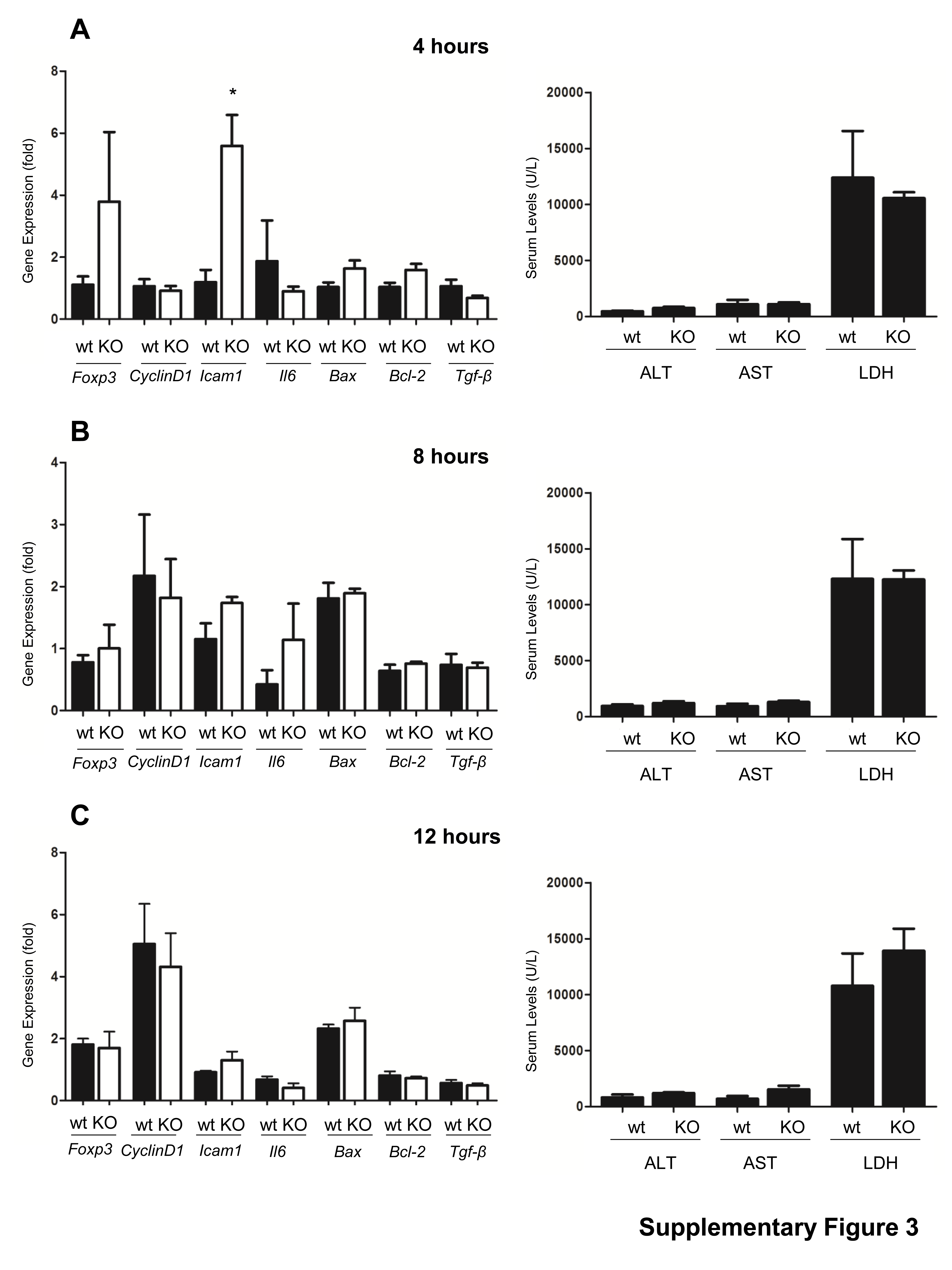

Supplement: S3 Fig — Hepatic gene expression and ALT, AST and LDH serum levels in wt and Ccr6-/- treated mice with clodronate liposome and single CCl4 injection at (A) 4 hours (n = 4 each group). (B) 8 hours (n = 4 each group) and (C) 12 hours (n = 4 wt mice, n = 5 Ccr6-/- mice) post CCl4 injection (*p<0.05). (TIF) [file pone.0145147.s004.tif]

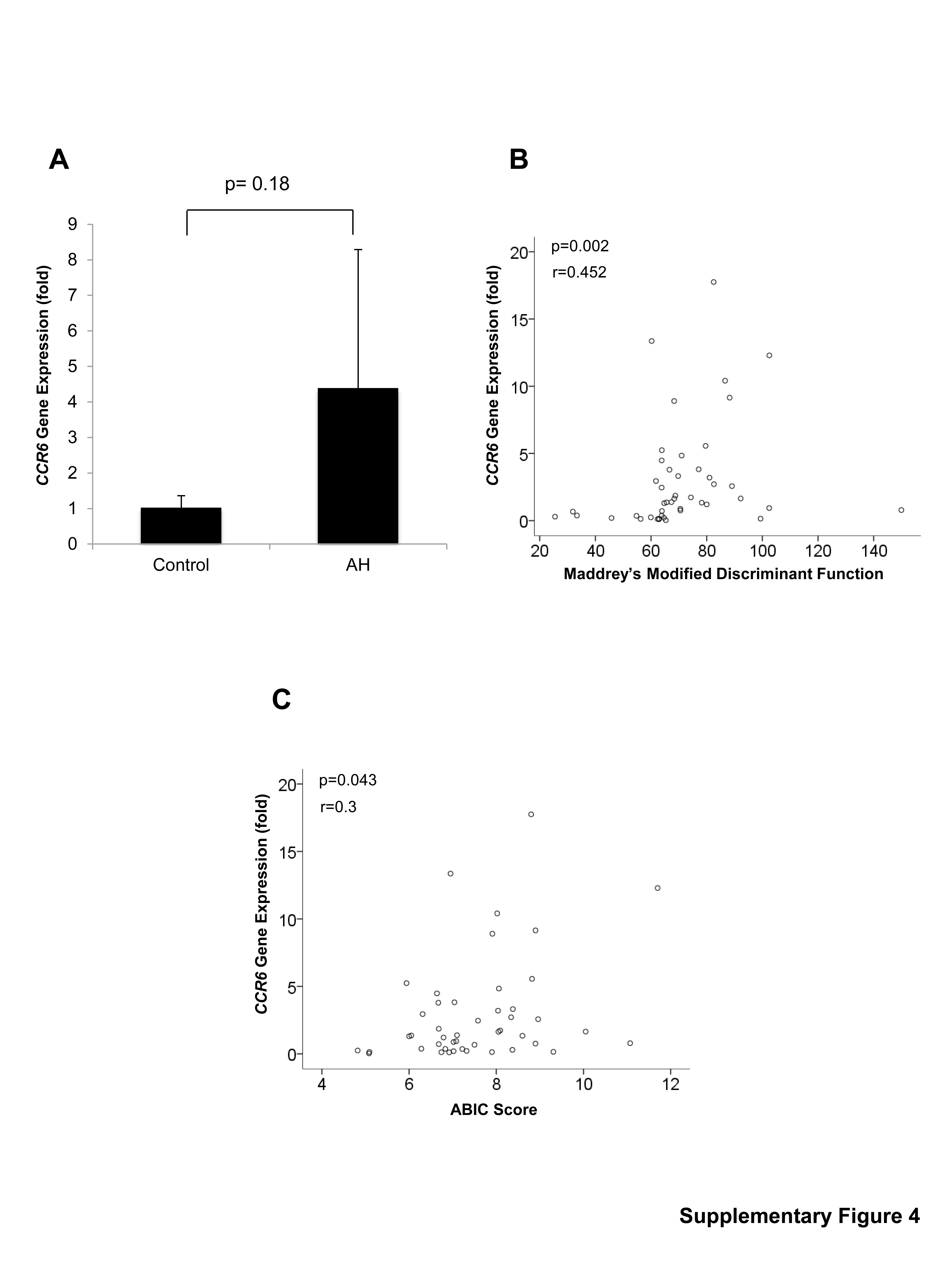

Supplement: S4 Fig — (A) CCR6 hepatic gene expression in patients with AH (n = 46). (B) Correlation between CCR6 hepatic gene expression and Maddrey’s modified discriminant function in patients with AH (n = 46) (p = 0.002); (C) Correlation between CCR6 hepatic gene expression and the ABIC score in patients with AH (n = 46) (p = 0.043). (TIF) [file pone.0145147.s005.tif]
