# Supplementary material for: Chemokine Receptor Ccr6 Deficiency Alters Hepatic Inflammatory Cell Recruitment and Promotes Liver Inflammation and Fibrosis
Source: PLoS One. 2015 Dec 21;10(12):e0145147. doi: 10.1371/journal.pone.0145147 (PMC4687007; doi:10.1371/journal.pone.0145147)
Supplement: S1 Text — (DOC) [file pone.0145147.s006.doc]

**Supplementary Materials**

**RNA isolation and PCR analysis**

RNA was isolated from human hepatic biopsies and from murine liver samples using Trizol (Invitrogen, Carlsbad, CA, USA) as previously described[1, 2]. After reverse transcription, mRNA levels were determined by quantitative real-time PCR on an ABI 7900HT cycler (Applied Biosystems, Life Technologies Corporation, Carlsbad, CA) using commercial primer-probe pairs (Applied Biosystems) for *Ccr6*, *Ccl20*, *Il6*, *Icam1*, *Tnf-α*, *Mcp1*, *Col1a1*, *Tgf-β*, *Il10*, *Mgl1*, *Il4*, *Il13*, *Gata3*, *Foxp3*, *CyclinD1*, *Bax* and *Bcl2*. Murine data were normalized to GAPDH and human data were normalized to 18s. The gene expression values were calculated based on the ∆∆Ct method and the results were expressed as 2-ΔΔCt.

**Histological assessment of polymorphonuclear cells in human biopsies**

Polymorphonuclear cells (PMN) assessment was blinded performed by en expert liver pathologist in human liver specimens from patients with AH as previously described [2, 3]. Briefly, liver specimens were formalin-fixed and paraffin-embebbed and 3 μm slices were stained with hematoxylin-eosin and Masson’s trichrome. We defined “mild” PMN infiltration the presence of isolated or a row of few PMNs around one or around a small cluster of 3-4 hepatocytes. We considered as “marked” PMN infiltration the presence of PMNs when they were easily recognized at low magnification (x200) and when we observed numerous PMN around damaged hepatocytes as previously described [3].

**Experimental models**

Male and female C57Bl/6 wild type (*wt*) (Charles River, l’Arbresle, France) and *Ccr6^-/-^* (The Jackson Laboratories, Bar Harbor, ME, USA) mice 8-12 weeks aged were administrated carbon tetrachloride (CCl_4_) intraperitoneally (i.p.) to induce acute or chronic liver injury. Mice were injected with 0.5-mL/kg body weight of vehicle (corn oil, Sigma-Aldrich, St. Louis, MO, USA) or CCl_4_ (Sigma-Aldrich) diluted 1:4 in corn oil. To induce acute liver injury, mice were injected once with CCl_4_ and were sacrificed 24 hours later. For the induction of liver fibrosis, CCl_4_ was injected three times a week for 2 or 4 weeks and mice were sacrificed 48 hours after the last injection. Macrophage depletion was performed by injecting mice with clodronate liposomes and as vehicle we used PBS liposomes (clodronateliposomes.org, Amsterdam, The Netherlands). 200 μl of clodronate or PBS liposomes were injected i.p. 24 hours before injecting mice with vehicle (corn oil) or CCl_4_ to induce an acute liver damage. Depletion efficiency was determined by immunohistochemistry, quantification of F4/80^+^ cells (10 random pictures for each mouse at magnification x200) and by quantitative real-time PCR. For time course experiment, 200 μl of clodronate or PBS liposomes were injected i.p. 24 hours before receiving a single injection of CCl_4_. Mice were sacrificed after 4 (n=4 each group), 8 (n=4 each group) and 12 hours (wt, n=4; Ccr6^-/-^, n=5).

All animal procedures were approved by the Ethics Committee of Animal Experimentation of the University of Barcelona and were conducted in accordance with the National Institutes of Health Guide for the Care and Use of Laboratory Animals and are in accordance with those set by the National Institutes of Health.

**Isolation of liver mononuclear cells and flow citometry analysis**

Liver mononuclear cells were isolated as previously described [4, 5] with some modifications. Briefly, livers were harvested from controls and CCl_4_ chronically injected mice, washed in PBS, pressed through a sterile 70 µm stainless steel mesh (BD Biosciences) and suspended in PBS as previously described [4, 5]. After washing, cells were resuspended in 33% Ficoll solution and centrifuged at 800 *g* for 20 minutes with brake off. Liver mononuclear cells were collected, washed and stained with F4/80-PE (eBioscience, Affymetrix, San Diego, CA, USA), CD11b-Alexa Fluor (Becton, Dickinson and Company, BD, New Jersey, NJ, USA), CD11c-FITC (BD), CD45R/B220-PE (BD), MHC Class II (I-A/I-E)-APC (eBioscience) and Th17/Treg phenotyping kit (BD). Dead cells were excluded by using the Live/Dead cell stain kit (Life Technologies, Eugene, Oregon). All samples were analyzed by flow cytometry (FACS Canto II; BD) and data were analyzed with BD FACSDIVA^TM^ Software (BD).

**Tissue Staining and Immunohistochemistry**

Mouse paraffin-embedded liver sections were incubated with primary F4/80 (1:200, Serotec, Oxford, UK), MPO (1:50, Abcam), Ki67 (1:50, Abcam) and CCL20 (1:200, Santa Cruz Biotechnology, Dallas, TX, USA) antibodies overnight at 4°C. Human frozen liver sections from alcohol-induced liver cirrhosis were incubated with primary CCR6 antibody (1:200, R&D Systems, Minneapolis, Minnesota, USA) overnight at 4ºC. After several washing, sections were incubated with secondary antibodies (Dako, Glostrup, Denmark) for 30 minutes at room temperature, stained with 3,3’-diaminobenzidine (DAB, Dako) and counterstained with hematoxylin. Sections were visualized at magnification x200 and F4/80-positive, myeloperoxidase (MPO)-positive and Ki67-positive area were quantified in 9 different fields for each section by histomorphometry. Results were expressed as % of positive-stained area. To assess the presence of liver fibrosis, liver specimens were stained with Sirius Red (Gurr-BDH Lab Supplies; Poole, England); quantified using a morphometric analysis as previously described [2] and results were expressed as % of positive-stained area.

[1] Affo S, Dominguez M, Lozano JJ, Sancho-Bru P, Rodrigo-Torres D, Morales-Ibanez O, et al. Transcriptome analysis identifies TNF superfamily receptors as potential therapeutic targets in alcoholic hepatitis. Gut 2013;62:452-460.

[2] Affo S, Morales-Ibanez O, Rodrigo-Torres D, Altamirano J, Blaya D, Dapito DH, et al. CCL20 mediates lipopolysaccharide induced liver injury and is a potential driver of inflammation and fibrosis in alcoholic hepatitis. Gut 2014;63:1782-1792.

[3] Altamirano J, Miquel R, Katoonizadeh A, Abraldes JG, Duarte-Rojo A, Louvet A, et al. A histologic scoring system for prognosis of patients with alcoholic hepatitis. Gastroenterology 2014;146:1231-1239 e1231-1236.

[4] Zheng SJ, Wang P, Tsabary G, Chen YH. Critical roles of TRAIL in hepatic cell death and hepatic inflammation. J Clin Invest 2004;113:58-64.

[5] Beraza N, Malato Y, Sander LE, Al-Masaoudi M, Freimuth J, Riethmacher D, et al. Hepatocyte-specific NEMO deletion promotes NK/NKT cell- and TRAIL-dependent liver damage. J Exp Med 2009;206:1727-1737.
